# Common variation in the sodium/glucose cotransporter 2 gene *SLC5A2* does neither affect fasting nor glucose-suppressed plasma glucagon concentrations

**DOI:** 10.1371/journal.pone.0177148

**Published:** 2017-05-04

**Authors:** Anna-Maria Ordelheide, Anja Böhm, Daniela Kempe-Teufel, Robert Wagner, Fausto Machicao, Martin Heni, Norbert Stefan, Andreas Fritsche, Hans-Ulrich Häring, Harald Staiger

**Affiliations:** 1 Institute for Diabetes Research and Metabolic Diseases of the Helmholtz Center Munich at the Eberhard Karls University Tübingen, Otfried-Müller-Straße 10, Tübingen, Germany; 2 German Center for Diabetes Research (DZD), Otfried-Müller-Straße 10, Tübingen, Germany; 3 Division of Endocrinology, Diabetology, Angiology, Nephrology, and Clinical Chemistry, Department of Internal Medicine, University Hospital Tübingen, Otfried-Müller-Straße 10, Tübingen, Germany; 4 Institute of Experimental Genetics, Helmholtz Center Munich, German Research Center for Environmental Health, Ingolstädter Landstraße 1, Neuherberg, Germany; 5 Division of Nutritional and Preventive Medicine, Department of Internal Medicine, University Hospital Tübingen, Otfried-Müller-Straße 10, Tübingen, Germany; 6 Interfaculty Center for Pharmacogenomics and Pharma Research at the Eberhard Karls University Tübingen, Auf der Morgenstelle 8, Tübingen, Germany; 7 Institute of Pharmaceutical Sciences, Department of Pharmacy and Biochemistry, Eberhard Karls University Tübingen, Auf der Morgenstelle 8, Tübingen, Germany; Consiglio Nazionale delle Ricerche, ITALY

## Abstract

**Aim:**

Inhibition of sodium/glucose cotransporter 2 (SGLT2), the key transport protein in renal glucose reabsorption, promotes glucose excretion and represents a new concept in the therapy of type-2 diabetes. In addition, SGLT2 inhibition elevates circulating glucagon concentrations and enhances hepatic glucose production. Since SGLT2 is expressed in human pancreatic α-cells and regulates glucagon release, we tested whether common variants of the SGLT2 gene *SLC5A2* associate with altered plasma glucagon concentrations in the fasting state and upon glucose challenge.

**Methods:**

A study population of 375 healthy subjects at increased risk for type-2 diabetes, phenotyped by a 5-point oral glucose tolerance test (OGTT) and genotyped for recently described *SLC5A2* tagging single nucleotide polymorphisms (SNPs), was selected for plasma glucagon measurements.

**Results:**

After adjustment for gender, age, body mass index, and insulin sensitivity, the four tagging SNPs (rs9924771, rs3116150, rs3813008, rs9934336), tested separately or as genetic score, were neither significantly nor nominally associated with plasma glucagon concentrations at any time during the OGTT, with the inverse AUC of glucagon or the glucagon fold-change during the OGTT (p ≥ 0.2, all). Testing for genotype-related differences in the time course of the glucagon response using MANOVA did also not reveal any significant or nominal associations (p ≥ 0.5, all).

**Conclusion:**

We could not obtain statistically significant evidence for a role of common *SLC5A2* variants in the regulation of glucagon release in the fasting state or upon glucose challenge. Moreover, the reported nominal effects of individual *SLC5A2* variants on fasting and post-challenge glucose levels may probably not be mediated by altered glucagon release.

## Introduction

Even though very well soluble in water, less than one percent of glucose filtered by the healthy kidney is excreted in the urine [1]. Two sodium-dependent glucose transporters, i.e., sodium/glucose cotransporter (SGLT) 1 and 2, are the major transport proteins responsible for renal glucose reabsorption: more than 90 percent of glucose is reabsorbed by SGLT2 and nearly three percent by SGLT1 [2]. Both are located in the proximal convoluted tubule of the nephron [3]. Only in the pathophysiological state of hyperglycemia, glucose is excreted by glucosuria due to substrate saturation of the SGLTs [2]. This renal glucose loss is however insufficient to normalize the elevated blood glucose levels of diabetic patients. Rather, the rise in glucose reabsorption up to the SGLTs’ transport maximum is considered to contribute to sustained hyperglycemia [1].

The concept to fight diabetes by enhancing glucose excretion via pharmacological inhibition of glucose reabsorption was stimulated by the characterization of the natural O-glucoside phlorizin, known for its glucosuria-inducing properties since more than 100 years [4], as competitive inhibitor of SGLT1 and SGLT2 [5]. The development of more selective SGLT2 inhibitors with less severe gastrointestinal side effects than phlorizin and its derivative T-1095 has become successful, and dapagliflozin, canagliflozin, and empagliflozin were recently approved for clinical use as anti-diabetic drugs.

Results from clinical trials with SGLT2 inhibitors in type-2 diabetic patients revealed not only efficient reductions in blood glucose concentrations but also increased hepatic glucose production [6,7]. In these studies, concomitant increases in fasting and mixed-meal-induced circulating glucagon concentrations were observed [6,7]. Concerning possibly underlying mechanisms, it was recently shown that SGLT2 expression is not restricted to the kidney, but is also found in glucagon-secreting α-cells of human pancreatic islets, and that SGLT2 inhibition triggers glucagon release from human islets via K_ATP_ channel activation [8].

Prompted by this novel role of SGLT2 in the regulation of glucagon release, we tested whether recently reported common genetic variation in the human SGLT2 gene *SLC5A2* [9] is associated with circulating glucagon concentrations during a 5-point oral glucose tolerance test (OGTT) in subjects at risk for type-2 diabetes.

## Materials and methods

### Ethics statement

All procedures were in accordance with the Ethical Principles for Medical Research Involving Human Subjects (Declaration of Helsinki) of the World Medical Association and were approved by the Ethics Committee of the Eberhard Karls University Tübingen. Informed written consent was obtained from all study participants.

### Study population

The ongoing Tübingen Family (TÜF) study for type-2 diabetes currently includes more than 2,800 non-diabetic individuals at increased risk for type-2 diabetes, having a family history of type-2 diabetes, a body mass index (BMI) ≥ 27 kg/m^2^, impaired fasting glycemia, and/or previous gestational diabetes [10]. All study participants were assessed for medical history, smoking status, and alcohol consumption habits, and underwent physical examination, routine blood tests, and oral glucose tolerance tests (OGTTs). In this at-risk population, 2,229 subjects who (i) donated DNA, (ii) had complete 5-point OGTT data sets, and (iii) had documented absence of medication modulating glucose tolerance, insulin sensitivity, or insulin secretion were successfully genotyped for *SLC5A2* variants in the course of a recent investigation [9]. From this well phenotyped and genotyped group, a study population of 375 subjects was selected on the basis of available plasma glucagon measurements. The clinical data of this study population are presented in Table 1. Less than 5% of the study participants were related. Four subjects were on anti-hyperlipidemic medication (three on statins and one on ezetimibe).

**Table 1:** Clinical data of the study population (N = 375).

Gender (women / men)	254 / 121
Age (y)	37.9 ± 12.3
BMI (kg/m^2^)	27.5 ± 6.6
Body fat content (%)	30.3 ± 9.8
NGT / IFG / IGT / IFG+IGT	277 / 34 / 42 / 22
Glucose, fasting (mmol/L)	5.10 ± 0.52
Glucose, 120 min OGTT (mmol/L)	6.17 ± 1.55
Glucose, AUC 0–120 min OGTT (mmol/L)	14.3 ± 2.9
Insulin, fasting (pmol/L)	64.3 ± 52.4
Insulin, 120 min OGTT (pmol/L)	423 ± 384
Insulin, AUC 0–120 min OGTT (pmol/L)	904 ± 667
Glucagon, fasting (pmol/L)	66.2 ± 24.6
Glucagon, 120 min OGTT (pmol/L)	53.6 ± 18.7
Glucagon, AUCi 0–120 min OGTT (pmol/L)	22.3 ± 24.3

Data are given as counts or means ± SD. AUC(i)–(inverse) area under the curve; BMI–body mass index; IFG–impaired fasting glycemia; IGT–impaired glucose tolerance; NGT–normal glucose tolerance; OGTT–oral glucose tolerance test

### Oral glucose tolerance test

A standardised 75-g OGTT was performed after a 10-h overnight fast. For the measurement of plasma glucose, serum insulin, and plasma glucagon concentrations, venous blood samples were drawn at baseline and 30, 60, 90, and 120 min after start of the OGTT as previously described [10].

### Assessment of body adiposity

BMI was calculated as weight divided by squared height (in kg/m^2^). The body fat content (in %) was measured by bioelectrical impedance (BIA-101, RJL Systems, Detroit, MI, USA).

### Laboratory measurements

Plasma glucose concentrations (in mmol/L) were measured with a bedside glucose analyser (glucose oxidase method, Yellow Springs Instruments, Yellow Springs, OH, USA). Serum insulin concentrations (in pmol/L) were determined with a commercial chemiluminescence assay for ADVIA Centaur (Siemens Medical Solutions, Fernwald, Germany). Plasma concentrations of triglycerides and total cholesterol (in mg/dL, both) were measured with the ADVIA 1800 Clinical Chemistry System (Siemens Healthcare Diagnostics, Eschborn, Germany). Glucagon concentrations (in pmol/L) were determined after one freeze-thaw cycle in plasma samples obtained from EDTA tubes containing aprotinin as protease inhibitor, and the measurements were performed by radioimmunoassay (Linco Research/Millipore, St. Charles, MO, USA).

### Calculations

The areas under the curves (AUCs) of glucose and insulin concentrations during the 5-point OGTT were determined using the trapezoid method: AUC = 0.5 * [0.5 * c(analyte)_0_ + c(analyte)_30_ + c(analyte)_60_ + c(analyte)_90_ + 0.5 * c(analyte)_120_] with c = concentration and subscript = time of measurement. The inverse AUC (AUCi) of glucagon concentrations during the OGTT was calculated as previously reported [11]: 0.5 * [0.5 * (|c(glucagon)_0_ –c(glucagon)_30_|)] + 0.5 * [0.5 * (|c(glucagon)_30_ –c(glucagon)_60_|) + c(glucagon)_0_ –c(glucagon)_30_] + 0.5 * [0.5 * (|c(glucagon)_60_ –c(glucagon)_90_|) + c(glucagon)_0_ –c(glucagon)_60_] + 0.5 * [0.5 * (|c(glucagon)_90_ –c(glucagon)_120_|) + c(glucagon)_0_ –c(glucagon)_90_]. Fold changes of glucagon concentrations during the OGTT were calculated as ratio c(glucagon)_120_ / c(glucagon)_0_. Insulin sensitivity was estimated from glucose and insulin concentrations during the OGTT according to Matsuda and DeFronzo [12]: 10,000 / [c(glucose)_0_ * c(insulin)_0_ * c(glucose)_mean_ * c(insulin)_mean_]^½^.

### SNP selection and genotyping

Selection of the tagging SNPs and their genotyping were recently described in detail [9]. In brief, the four common *SLC5A2* SNPs, i.e., rs9924771 G/A and rs9934336 G/A in intron 1 and rs3813008 G/A and rs3116150 G/A in intron 5, were identified as tagging SNPs by *in silico* analysis of publicly available linkage disequilibrium data from the 1000 Genomes Project (CEU population). For genotyping, DNA was extracted from whole blood, and DNA sequences harbouring the SNPs were amplified by polymerase chain reactions. SNPs rs9934336, rs3813008, and rs3116150 were genotyped by mass spectrometry (massARRAY, Sequenom, Hamburg, Germany). SNP rs9924771 was genotyped by allelic discrimination using a TaqMan assay (Applied Biosystems, Foster City, CA, USA).

### Statistical analyses

Prior to statistical evaluation, continuous variables with skewed distribution were log_*e*_-transformed to approximate normal distribution. For multiple linear regression analysis, the least-squares method was applied and the trait of interest (glucagon concentration, AUC, or fold-change) was used as dependent variable, the SNP genotype (in the additive or dominant inheritance model, respectively) as independent variable, and gender, age, and BMI as confounding variables. Differences between genotypes in the time course of the glucagon response, independent of the mentioned confounders, were tested by multivariate analysis of variance (MANOVA). To correct for the four SNPs tested in parallel, a study-wide significance threshold of p < 0.0127 was chosen according to Bonferroni correction for multiple comparisons. For these analyses as well as for power calculations, the statistical software package JMP 10.0 (SAS Institute, Cary, NC, USA) was used.

## Results

As depicted in Table 1, the study population consisted to 68% of female subjects. On average, the population was middle-aged (38 years old) and overweight (BMI 27.5 kg/m^2^). The subjects were to 74% of normal glucose tolerance (26% prediabetic).

The SNP effects on glucagon concentrations were analysed in the additive and dominant inheritance models. Associations were tested by multiple linear regression models with gender, age, BMI, and insulin sensitivity as covariates. In these fully adjusted regression models, testing the effects of the least frequent SNP, i.e., rs3813008, on glucagon concentrations in the additive inheritance model revealed that the study was sufficiently powered (1-β > 0.8) to detect effect sizes ≥ 5% at a nominal α-threshold of 0.05.

At any time of the 5-point OGTT analysed, none of the four tagging SNPs (rs9924771, rs3116150, rs3813008, rs9934336) was significantly (p < 0.0127) or nominally (p < 0.05) associated with plasma glucagon concentrations (p ≥ 0.2, all; Table 2). The same holds for the SNPs’ associations with the AUCi of glucagon and the glucagon fold-change during the OGTT (p ≥ 0.2, all; Table 2). Testing for differences between the SNP genotypes in the time course of the glucagon response using MANOVA to adjust for the aforementioned confounders did also not reveal any significant or nominal associations (p ≥ 0.5, all). Inclusion of plasma triglyceride and total cholesterol concentrations as well as anti-hyperlipidemic medication as additional confounding variables in the regression models and in the MANOVA did not result in any significant or nominal associations between the SNPs and the glucagon measurements (p ≥ 0.2, all).

**Table 2:** Associations of *SLC5A2* SNPs with serum glucagon concentrations during the 5-point OGTT.

	Geno-type	N	Glucagon 0 min (pmol/L)	Glucagon 30 min (pmol/L)	Glucagon 60 min (pmol/L)	Glucagon 90 min (pmol/L)	Glucagon 120 min (pmol/L)	Glucagon AUCi 0–120 min (pmol/L)	Glucagon decrease 0–120 min (fold-change)
rs9924771	GG	181	65.6 ± 25.0	62.4 ± 21.9	56.5 ± 21.2	53.5 ± 18.4	53.3 ± 19.3	21.4 ± 25.2	0.86 ± 0.27
	GA	157	67.5 ± 25.0	61.8 ± 23.0	56.3 ± 21.1	53.7 ± 20.0	54.1 ± 18.9	24.6 ± 24.4	0.84 ± 0.25
	AA	37	63.4 ± 20.4	63.5 ± 19.9	56.2 ± 20.0	54.7 ± 16.3	53.4 ± 14.9	16.7 ± 18.2	0.89 ± 0.24
p_add_ / p_dom_	-	-	0.7 / 0.9	0.7 / 0.6	0.9 / 1.0	0.9 / 1.0	0.7 / 0.6	0.5 / 0.9	0.3 / 0.6
rs3116150	GG	211	66.0 ± 24.0	61.9 ± 21.0	56.1 ± 20.9	54.0 ± 19.1	53.8 ± 18.4	22.3 ± 25.5	0.86 ± 0.27
	GA	141	67.3 ± 25.8	63.5 ± 24.1	56.8 ± 21.3	53.9 ± 18.6	53.6 ± 18.8	22.9 ± 22.8	0.83 ± 0.23
	AA	23	60.6 ± 22.7	57.7 ± 20.0	55.5 ± 20.7	49.8 ± 18.2	52.6 ± 21.0	18.7 ± 23.8	0.92 ± 0.30
p_add_ / p_dom_	-	-	0.8 / 0.8	0.8 / 0.9	0.7 / 0.7	0.5 / 0.8	0.7 / 0.8	1.0 / 0.8	0.9 / 0.5
rs3813008	GG	274	65.6 ± 24.5	61.8 ± 21.7	55.7 ± 20.5	53.5 ± 18.1	53.3 ± 18.5	22.1 ± 25.5	0.86 ± 0.26
	GA	96	68.6 ± 25.2	63.9 ± 23.6	58.4 ± 22.6	54.3 ± 21.2	54.8 ± 19.6	24.0 ± 20.6	0.84 ± 0.25
	AA	5	51.4 ± 11.6	57.3 ± 16.7	51.9 ± 16.6	56.5 ± 16.4	52.4 ± 15.1	-0.2 ± 8.7	1.01 ± 0.07
p_add_ / p_dom_	-	-	0.8 / 0.5	0.6 / 0.5	0.3 / 0.2	0.8 / 0.8	0.4 / 0.4	0.5 / 1.0	0.5 / 0.8
rs9934336	GG	203	66.2 ± 24.4	62.4 ± 22.4	56.4 ± 20.5	54.2 ± 18.4	54.4 ± 18.6	21.8 ± 22.7	0.86 ± 0.25
	GA	155	66.1 ± 25.2	61.9 ± 22.3	56.2 ± 22.2	52.6 ± 19.7	52.8 ± 18.9	23.3 ± 26.1	0.85 ± 0.26
	AA	17	66.1 ± 21.9	63.2 ± 17.6	57.1 ± 15.6	58.7 ± 15.6	51.8 ± 17.6	18.3 ± 27.1	0.82 ± 0.32
p_add_ / p_dom_	-	-	0.9 / 1.0	0.8 / 1.0	1.0 / 0.7	0.9 / 0.4	0.4 / 0.3	0.8 / 0.5	0.2 / 0.2

Glucagon concentrations are shown as unadjusted raw data (means ± SD). Prior to statistical analysis, non-normally distributed data were log_*e*_-transformed. Associations between SNP genotypes and glucagon concentrations were tested by multiple linear regression analysis (standard least squares method) with gender, age, BMI, and insulin sensitivity as covariates. All SNPs were analysed in the additive and dominant inheritance models (p_add /_ p_dom_). AUCi–inverse area under the curve; OGTT–oral glucose tolerance test; SNP–single nucleotide polymorphism

It appears noteworthy that, even though not significantly different from the other genotype groups, the rs3813008 AA genotype group (consisting of five subjects only) displayed markedly lower fasting glucagon concentrations (Table 2). Moreover, the glucagon levels did not decrease during the OGTT in this genotype group (Table 2).

To see whether allele summation can reveal significant associations of the *SLC5A2* gene with glucagon concentrations, we generated simple unweighted genetic scores based on information from multiple linear regression analysis about the direction of the SNPs’ effect estimates for association with fasting glucagon. In the additive inheritance model with gender, age, BMI, and insulin sensitivity as covariates, the major alleles of rs9924771 and rs3116150 and the minor alleles of rs3813008 and rs9934336 generated positive effect estimates indicating (marginal and non-significant) glucagon-elevating effects. The score summing up these alleles was neither significantly nor nominally associated with the aforementioned glucagon measurements in the fully adjusted multiple linear regression analyses (p ≥ 0.5) or in the MANOVA (p = 0.7). In the dominant inheritance model including the same covariates, the minor alleles of all four SNPs generated positive effect estimates. Summing up all minor alleles did not show significant associations of *SLC5A2* with glucagon measurements (p ≥ 0.4, multiple linear regression; p = 0.6, MANOVA).

## Discussion

According to molecular *in vitro* and *in vivo* studies, SGLT2 is involved in the regulation of glucagon secretion from pancreatic α-cells [8,13]. Therefore, we analysed whether common genetic variation in the human SGLT2 gene *SLC5A2* associates with fasting and glucose-suppressed circulating glucagon concentrations. We analysed the four tagging SNPs that were recently tested in a larger study population (N = 2,229) from our TÜF study for association with glycemic traits, insulin sensitivity, insulin secretion, blood pressure, and glomerular filtration [9]. In this earlier study, we found nominal associations of SNP rs3116150 with fasting glycemia, glucose excursions during the 5-point OGTT (AUC glucose), and systolic blood pressure [9]. Three of our four SNPs (rs3116150, rs3813008, rs9934336) were also studied by Enigk *et al*. for association with glucose and insulin concentrations during a 3-point OGTT in Sorbs (N = 1,013) and a 5-point OGTT in the Metabolic Syndrome Berlin Potsdam study (N = 2,042) [14]. These authors described nominal associations of SNP rs9934336 with glucose excursions during the OGTT in Sorbs as well as in the meta-analysis of both studies [14]. Thus, there was, at the beginning of this investigation, evidence that two of the tagging SNPs (i.e., rs3116150 and rs9934336) may exert effects *in vivo*.

The four tagging SNPs, tested separately or as genetic score, were however neither significantly nor nominally associated with glucagon concentrations at discrete times of the OGTT, with AUCi of glucagon, glucagon fold-changes, or with time courses of the glucagon response during the 5-point OGTT. Furthermore, we could demonstrate that these negative results are not due to limited statistical power as our study had sufficient statistical power to detect effect sizes ≥ 5% in the fully adjusted regression models. Clearly, nominal or even significant SNP effect sizes < 5% cannot be excluded due to the statistical limitations of our study’s sample size. Therefore, we encourage genetic replication efforts in similarly phenotyped and, if possible, larger studies having DNA and protease-inhibitor-treated plasma samples for glucagon measurements available. In particular, replication studies may help clarify whether the markedly reduced fasting glucagon concentrations and the lack of response to oral glucose in the five rs3813008 AA-allele carriers are real. If so, this genotype may emerge as a determinant contributing to the recently described phenotype of non-suppressed glucagon release upon glucose challenge, a phenotype that was associated with lower body weight and liver fat content, higher insulin sensitivity, and a reduced risk of glucose intolerance [15].

In conclusion, we could not obtain statistically significant evidence for a role of common variation affecting the *SLC5A2* gene (encoding SGLT2) in the regulation of glucagon release in the fasting state or upon glucose challenge. Moreover, the reported nominal effects of individual *SLC5A2* variants on fasting and post-challenge glucose levels [9,14] may probably not be mediated by altered glucagon release.
